# 不同转移潜能人大细胞肺癌细胞株转移相关microRNAs的筛选研究

**DOI:** 10.3779/j.issn.1009-3419.2011.11.01

**Published:** 2011-11-20

**Authors:** 为山 鲁, 书军 李, 彬 刘, 洋 李, 猛 罗, 丽亚 孙, 嘉琮 尤, 清华 周

**Affiliations:** 1 610041 成都，四川大学华西医院 West China Hospital, Sichuan University, Chengdu 610041, China; 2 300052 天津，天津医科大学总医院，天津市肺癌研究所，天津市肺癌转移与肿瘤微环境重点实验室 Tianjin Key Labotatory of Lung Cancer Metastasis and Tumor Microenvironment, Tianjin Lung Cancer Institute, Tianjin Medical University General Hospital, Tianjin 300052, China

**Keywords:** 肺肿瘤, 细胞系, 转移, 微小RNA, Lung neoplasms, Cell line, Metastasis, MicroRNAs

## Abstract

**背景与目的:**

微小RNA（microRNAs, miRNAs）参与调节肿瘤发生发展的多个过程，包括细胞的分裂增殖、细胞周期、凋亡、血管形成、侵袭和转移等。本研究应用miRNA芯片检测具有高低不同转移潜能人大细胞肺癌细胞株L9981和NL9980的miRNA表达谱，从中筛选出与大细胞肺癌转移相关的miRNAs。

**方法:**

收集L9981和NL9980细胞，抽提总RNA进行CY3标记，将标记RNA在miRNA芯片上进行杂交反应。通过数据统计分析，筛选出表达明显差异的miRNAs。应用Real-time PCR验证芯片结果，并应用生物信息学方法预测靶基因。

**结果:**

在不同转移潜能人大细胞肺癌L9981和NL9980细胞株中共筛选到22个表达明显差异的miRNAs。与NL9980相比，在L9981中有13个miRNAs表达上调，9个表达下调。Real-time PCR验证miR-125a-3p在细胞中的表达水平与芯片结果趋势一致，预测其靶基因可能为胰岛素样生长因子2。

**结论:**

筛选得到与大细胞肺癌转移相关的miRNA表达谱。

## Abstract (en)

**Background and objective:**

MicroRNAs (miRNAs) regulate a wide range of cancer-associated processes, including cell division, proliferation, cell cycle, apoptosis, angiogenesis, invasion, and metastasis. A microarray was performed to analyze metastasis-related miRNAs with different metastastic potentials and to further elucidate their mechanism in the large-cell lung cancer cell lines.

**Methods:**

L9981 and NL9980 cells were harvested, and total RNA was extracted for CY3. RNA hybridization was then performed on the chip with marked miRNAs. MiRNAs with significantly different expression were selected through statistical analysis. A real-time polymerase chain reaction (PCR) was employed to validate the results of the microarray, and target genes were predicted using bioinformatics.

**Results:**

Expressions of 22 miRNAs were significantly different in the L9981/NL9980 cell lines. Compared with the NL9980, 13 miRNAs were upregulated in the L9981 cell lines, whereas 9 miRNAs were downregulated. The result of miR-125a-3p expression based on real-time PCR was consistent with the microarray. Insulin-like growth factor 2 might be a target gene of miR-125a-3p.

**Conclusion:**

The metastatic miRNA profile in large-cell lung cancer was successfully screened out.

肺癌是全球发病率和病死率最高的恶性肿瘤，约占所有肿瘤的25%，且发病率呈上升趋势^[1]^。临床资料显示，多数患者在就诊时就已经有局部浸润和远处转移，在肿瘤完全切除之后仍出现肺癌的复发和转移。非小细胞肺癌（non-small cell lung cancer, NSCLC）包括鳞癌、腺癌、大细胞癌，约占肺癌总数的80%-85%。因此，研究大细胞肺癌转移机制和早期分子标志，对于NSCLC的诊断及个体化治疗具有重要意义。

微小RNA（microRNAs, miRNAs）是一类非编码单链小RNA，长度约为21 nt-23 nt，通过与目标mRNAs互补配对导致mRNAs降解或抑制转录后翻译诱导基因沉默^[2]^。有研究^[3-5]^表明，体内可能存在与蛋白信号通路网络类似的miRNAs网络，而miRNAs网络与EMT过程、肿瘤血管生成、肿瘤转移的表观遗传修饰等过程有着密切的联系。因此，通过miRNA芯片筛选不同转移潜能人大细胞肺癌细胞株中转移相关miRNAs的差异表达，有助于进一步研究miRNAs在NSCLC侵袭转移中的分子机制，为阻断NSCLC侵袭转移的信号通道和逆转NSCLC侵袭转移表型提供新的靶点和途径。

## 1 材料与方法

### 1.1 细胞株

高转移人肺大细胞癌细胞株L9981和低转移人肺大细胞癌细胞株NL9980是周清华教授从人大细胞肺癌细胞系WCQH-9801母系细胞中分离建立起来的，它们具有相同遗传学背景、不同的侵袭能力和分子生物学特性^[6, 7]^（由天津市肺癌研究所，天津市肺癌转移与肿瘤微环境重点实验室提供）。用含10%小牛血清（FBS, GIBCO）的RPMI1640（Invitrogen, Carlsbad, CA），置于37 ℃、5%CO_2_孵箱中培养。

### 1.2 主要试剂

MiRNA Complete Labeling and Hyb Kit、Gene Expression Wash Buffer Kit和Human miRNA microarray购自Agilent公司，mirVana RNA Isolation Kit购自Ambion公司，RNase-Free DNase Set(50)购自Qiagen公司，反转录酶M-MLV Reverse Transcriptase购自Promega公司，RNA酶抑制剂购自Takara公司，荧光定量PCR试剂SYBR^®^RPremix Ex Taq^TM^购自Takara公司。

### 1.3 miRNA芯片检测L9981和NL9980的miRNA表达谱

#### 1.3.1 细胞总RNA提取

当细胞处于对数生长期时收集细胞，按mirVana RNA Isolation Kit说明书提取总RNA。利用紫外分光光度计对总RNA进行浓度和纯度鉴定，1%凝胶电泳鉴定其完整性。

#### 1.3.2 芯片杂交

用Agilent’s miRNA Complete Labeling and Hyb Kit进行RNA杂交和洗染。用Rnase-free水将RNA样品稀释至50 ng/μL。取2 μL样品加入去磷酸化混合液总体积至4 μL，混匀后置于37 ℃金属浴中，保温30 min。在每管样品中添加2.8 μL 100%DMSO，置于100 ℃金属浴中加热5 min-10 min。反应结束将样品迅速转入冰水浴中冷却。取连接反应混合液4.5 μL加入样品管中混匀，稍离心，置于16 ℃温育2 h。然后将样品置于真空浓缩仪中完全抽干备用。将抽干的样品重新溶解在18 μL nuclease-free水中，每管加入4.5 μL配制好的10×GE Blocking Agent，加入22.5 μL的2×Hi-RPM Hybridization Buffer，混匀，置于100 ℃金属浴中加热5 min。反应结束后，迅速将其转至冰水浴中冷却5 min。将合适的盖片放置在Agilent SureHyb chamber底座上。将约45 μL反应液吸至盖片上，将芯片点样面朝下放在盖片上，组装SureHyb chamber，并拧紧。晃动组装好的SureHyb chamber使里面所有气泡可以自由移动。将SureHyb chamber放置在杂交炉上，55 ℃、20 rpm杂交20 h。

#### 1.3.3 芯片洗染

从杂交炉中取出Hybridization chamber assembly，小心取下芯片/盖片，芯片面朝上，浸入Gene Expression Wash Buffer 1中，取下盖片，迅速将芯片放在slide-staining dish 2的slide rack中。开启磁力搅拌器，中速搅拌5 min。取出放有芯片的slide rack，吸水纸轻轻吸去上面沾染的液体，放入slide-staining dish 3中。开启磁力搅拌器，中速搅拌5 min，温度37 ℃。取出slide rack，吸水纸吸去上面沾染的液体。

#### 1.3.4 芯片扫描

芯片结果采用Agilent扫描仪进行扫描。将芯片放在slide holder中，点样面朝下，放入扫描仪进行扫描。

#### 1.3.5 数据处理

采用Feature Extraction进行处理分析。差异基因筛选标准：单色芯片：Fold Change≥1.5，flags不全为A的基因。Regulation=up为上调，Regulation=down为下调。双色芯片：Log_2_Ratio≥1或≤-1，flags不为A的基因。

### 1.4 Real-time PCR验证miR-125a-3p在L9981和NL9980中的相对表达

L9981和NL9980细胞总RNA提取同1.3.1。参照Promega反转录酶说明书取1 μg总RNA为模板进行miR-125a-3p mRNA逆转录。RT-PCR引物：miR-125a-3p：GTCGTATCCAGTGCAGGGTCCGAGGTATTCGCACTGGATACGACGGCTC，U6：GTCGTATCCAGTGCAGGGTCCGAGGTATTCGCACTGGATACGACAAAATATGGAAC。以cDNA为模板，参照Takara公司的SYBR^®^Premix Ex Taq^TM^说明书进行Real-time PCR，每个样本3个复孔。Real-time PCR引物序列如下：miR-125a-3p forward primer：GGCTACAGGTGAGGTTCTTG，U6 forward primer：TGCGGGTGCTCGCTTCGGCAGC，common reverse primer：CAGTGCAGGGTCCGAGGT。反应条件：95 ℃，10 s，1个循环; 95 ℃，5 s，60 ℃，34 s，40个循环。

### 1.5 靶基因预测

应用互联网上miRNAs靶基因预测软件（TargetScan, PicTar, miRanda）在线服务站点，预测差异表达miRNAs的靶基因，取3个软件预测到的基因作为靶基因。

### 1.6 统计学处理

采用SPSS 13.0软件进行统计学分析。实验数据以Mean±SD表示，统计学方法为*t*检验，*P* < 0.05为有统计学差异。

## 2 结果

### 2.1 细胞总RNA的定量和完整性分析

应用紫外分光光度计对总RNA纯度和浓度进行分析，结果显示，L9981和NL9980总RNA的D_260_/D_280_值均在1.9-2.1之间（表 1）。1%琼脂糖凝胶电泳结果显示，28S、18S和5S三条带均清晰、完整，28S和18S条带亮度比值约为2:1（图 1），表明提取的总RNA无降解、无蛋白污染。

**1:** MiRNA定量检测结果：L9981和NL9980总RNA的D_260_/D_280_值均在1.9-2.1之间 Information of miRNA samples: L9981 and NL9980 total RNA D_260_/D_280_ values were between 1.9-2.1

Cell lines	Volume (μL)	Concentration (μg/μL)	Total (μg)	A_260_/ A_280_	28S/18S
NL9980	50	0.626	31.3	2.08	1.6
NL9980	50	0.442	22.1	2.02	1.6
NL9980	50	0.661	33.0	2.08	1.6
L9981	50	0.523	26.2	2.08	1.6
L9981	50	0.582	29.1	2.08	1.7
L9981	50	0.519	26.0	2.08	1.8

**1:**
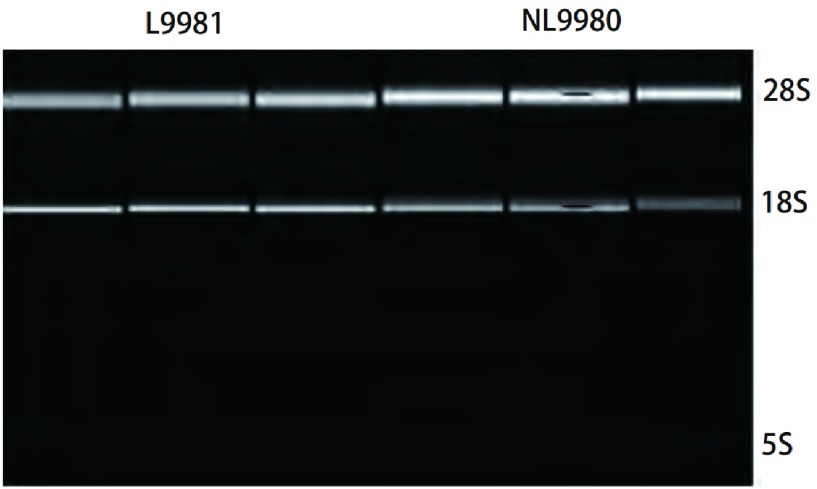
1%琼脂糖凝胶电泳结果：L9981和NL9980细胞总RNA的28S、18S和5S三条带均清晰、完整。 Electrophoresis of total RNA samples: L9981 and NL9980 total RNA, 28S, 18S and 5S three bands are clear and complete.

### 2.2 L9981和NL9980细胞株中差异表达的miRNAs

将L9981和NL9980细胞分别进行miRNA芯片检测，芯片杂交结果的扫描图如图 2所示。芯片结果经数据处理和统计学分析，结果显示，在具有高低不同转移潜能人大细胞肺癌L9981和NL9980细胞株中，共筛选到22个表达水平具有明显差异的miRNAs。与NL9980细胞相比，在L9981中有13个miRNAs表达水平上调（表 2），有9个miRNAs表达水平下调（表 3）。

**2:**
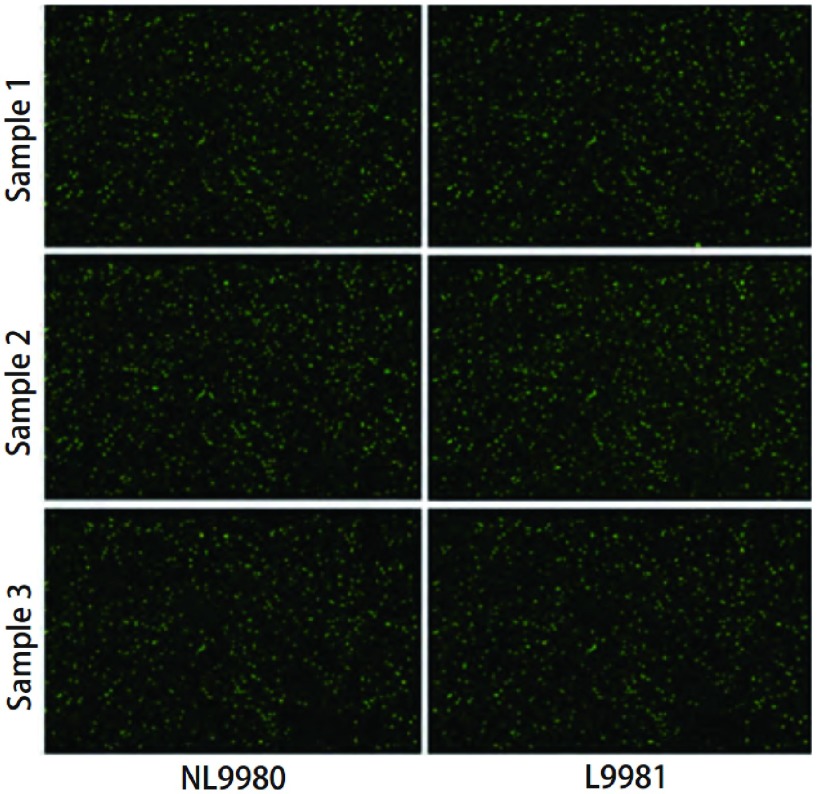
芯片杂交扫描图：在miRNA芯片上进行L9981/NL9980 RNA的杂交，每个样本重复3次。 Microarray scanning images of hybridization: RNAs of L9981/NL9980 cell lines were hybridized on microarray, each sample repeated three times.

**2:** 与NL9980细胞相比，在L9981细胞中有13个miRNAs表达上调 There are 13 miRNAs up regulated in L9981 cell lines

MiRNA	Fold（L9981/NL9980）
hsa-let-7e	2.254
hsa-miR-222	1.996
hsa-miR-130b	1.833
hsa-miR-487a	1.656
hsa-miR-222	1.599
hsa-miR-299-5p	1.555
hsa-miR-381	1.548
hsa-miR-598	1.547
hsa-miR-221	1.536
hsa-miR-380	1.533
hsa-miR-18b	1.526
hsa-miR-18a	1.511
hsa-miR-17	1.501

**3:** 与NL9980细胞相比，在L9981细胞中有9个miRNAs表达下调 There are 9 miRNAs down regulated in L9981 cell lines

MiRNA	Fold(L9981/NL9980)
hsa-miR-125a-3p	0.622
hsa-miR-126	0.627
hsa-miR-1202	0.629
hsa-miR-133a	0.633
hsa-miR-663	0.644
hsa-miR-183	0.652
hsa-miR-151-5p	0.654
hsa-miR-874	0.657
hsa-miR-31	0.658

### 2.3 miR-125a-3p在L9981/NL9980肺癌细胞系中的相对表达水平

我们应用Real-time PCR方法检测了miR-125a-3p在L9981和NL9980细胞株中的表达水平。实验结果显示，miR-125a-3p在高转移人肺大细胞癌细胞株L9981中的表达水平明显低于在低转移人肺大细胞癌细胞株NL9980中的表达水平（0.476±0.053 *vs* 1.000±0.096, *P*=0.004），提示miR-125a-3p可能具有抑制肿瘤转移的作用。

### 2.4 miR-125a-3p靶基因的预测

我们用3种常用的生物信息学软件TargetScan、PicTar和miRanda预测了miR-125a-3p的靶基因，发现胰岛素样生长因子2（insulin-like growth factors 2, IGF2）在3种软件中都能够被预测到，而且miR-125a-3p在IGF2的3’非编码区（3’untranslated regions, 3’UTRs）区有2个结合位点（图 3），因此IGF2可能为miR-125a-3p的靶基因。

**3:**
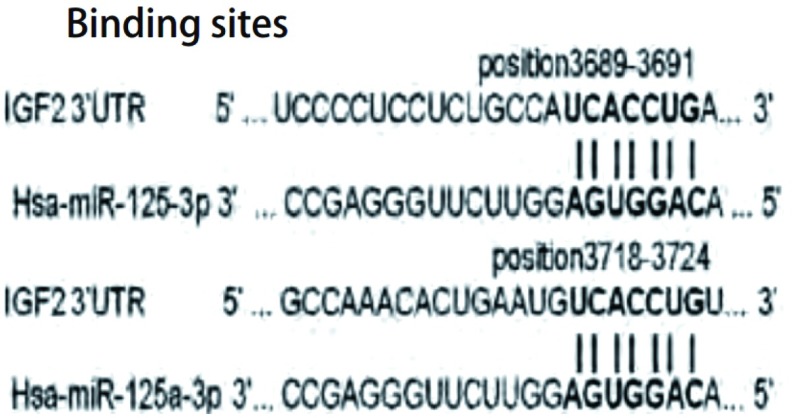
MiR-125a-3p在IGF2的3’UTR区有两个结合位点：一个位于IGF2的3’UTR的第3, 689个碱基-第3, 691个碱基，另一个位于IGF2的3’UTR的第3, 718个碱基-第3, 724个碱基。 Binding sites of miR-125a-3p in the 3'UTR of IGF2: there are two binding sites in the 3'UTR of IGF2 (one is at position of 3, 689-3, 691, another is at position of 3, 718-3, 724). IGF2: insulin-like growth factors 2; UTR: untranslated regions.

## 3 讨论

MiRNAs是一类非编码的小RNAs，长度大约为21 nt-23 nt，它们能够与靶基因mRNAs的3’UTR结合，在转录后水平上调控蛋白的表达^[2]^。大约50%的人miRNAs基因位于染色体脆性区，推测可能通过扩增、缺失或易位在肿瘤发生发展过程中发挥效应^[8]^。MiRNAs参与调节肿瘤发生的多个过程，其中包括细胞的分裂增殖、细胞周期、凋亡、血管形成。此外，越来越多的研究表明miRNAs在人类肿瘤的侵袭转移中起作用。

研究^[9]^发现，miR-200c和miR-200b能通过锌指E-盒结合同源异形盒（zinc-finger E-box binding homeobox, ZEB）1和2上调E-cadherin表达从而抑制上皮-间质转化（epithelial-mesenchymal transition, EMT）。有趣的是，ZEB1和ZEB2也能够通过与miR-200家族通用启动子的E-box或Z-box结合抑制miR-200家族的转录^[10]^。因此，在miR-200家族和ZEB1/ZEB2之间就形成了负反馈调节环路来保持EMT和间质-上皮转化（mesenchymal-epithelial transition, MET）之间的平衡^[11]^。MiRNA在肿瘤中也能起类似癌基因/抑癌基因的作用。MiR-10b、miR-21、miR-373、miR-378和miR-17-92在乳腺癌中能够促进肿瘤转移^[4, 12-15]^。而有些miRNAs能够抑制肿瘤转移，miR-335、miR-206和let-7家族能够在乳腺癌中起抑制肿瘤转移的作用^[16-18]^。虽然，目前对于肺癌转移相关miRNAs的研究较多，肺癌转移相关miRNAs表达谱以及miRNA在肺癌转移中的分子机制尚不清楚。

MiRNA芯片技术是高通量快速分析miRNAs表达谱的方法。L9981和NL9980细胞是周清华教授从人大细胞肺癌细胞系WCQH-9801母系细胞中分离构建出来的具有不同侵袭能力和分子生物学特性的两个肺癌细胞株，是具有相同遗传学背景的共源细胞株，具有高度的可比性。为了研究NSCLC转移相关miRNAs，我们应用miRNA芯片技术筛选了L9981与NL9980中差异表达的miRNAs。

我们在L9981和NL9980细胞株中共初步筛选到22个表达水平具有明显差异的miRNAs。与NL9980细胞相比，在L9981中有13个miRNAs表达水平上调，有9个miRNAs表达水平下调。其中miR-125a-3p在NL9980中的表达是L9981的1.6倍。我们又应用Real-time PCR方法检测了miR-125a-3p在L9981和NL9980细胞中的表达水平，检测结果与芯片结果趋势一致，从而也表明了芯片结果的可靠性。

MiR-125a是一种miRNA，在 http://microrna.sanger.ac.uk 数据库中发布了miR-125a的两种成熟体：has-miR-125a-3p和has-miR-125a-5p。已有研究^[19]^发现miR-125a-3p在NSCLC中表达下调，并且表达水平下调后可抑制NSCLC细胞的迁移和侵袭，提示miR-125a-3p在NSCLC细胞中具有抑制肿瘤转移的作用。这与我们的研究结果是相吻合的。此外，我们的实验结果还显示miR-126和miR-183在高转移人肺大细胞癌细胞株L9981中低表达，而miR-221和miR-222在高转移人肺大细胞癌细胞株L9981中高表达。

已有研究^[20]^发现，在肺癌细胞系中miR-126表达下调，而上调miR-126的表达能够抑制血管内皮细胞生长因子A（vascular endothelial growth factor A, VEGF-A)，在体内和体外上调miR-126能够抑制肺癌细胞的增殖。推测miR-126可能通过VEGF-A在肺癌中起抑制肿瘤增殖的作用。在肺癌细胞系中miR-126过表达可导致信号接头蛋白Crk蛋白表达水平降低，从而抑制肺癌细胞的粘附、迁移和侵袭能力^[21]^。另外研究^[22]^显示，miR-183的表达水平与肺癌细胞转移潜能负相关，而VIL2编码蛋白Ezrin是miR-183的靶点。提示miR-183可能通过Ezrin抑制肺癌细胞的转移潜能。而miR-221和miR-222在侵袭性NSCLC和肝细胞癌中的表达水平明显高于低侵袭性或正常的肺和肝细胞。MiR-221和miR-222以肿瘤抑制因子PTEN和TIMP3为靶点，诱导肿瘤坏死因子相关诱导配体（TNF-related apoptosis-inducing ligand, TRAIL）抵抗，并通过激活AKT通路和金属蛋白酶促进细胞迁移。此外，癌基因*MET*可通过调控c-Jun转录因子激活miR-221和miR-222的表达^[23]^。提示miR-221和miR-222可能具有促进肿瘤转移的作用。

MiRNAs通过与它们靶mRNAs的3’UTR区根据碱基互补配对原则结合，剪切或沉默靶mRNAs，从而达到转录后抑制的作用。要认识miRNAs的作用机制，需要认识miRNAs和靶基因的相互作用。研究者在研究过程中发现，miRNAs和靶基因的作用具有规律性，可以通过软件来预测。TargetScan、PicTar和miRanda是目前常用3种预测miRNAs靶基因的生物信息学软件^[24-26]^。我们用这3种软件预测了miR-125a-3p的靶基因。发现IGF2在这3个软件中都能被预测到，而且IGF2的3’UTR区有两个miR-125a-3p的结合位点。因此*IGF2*最有可能是miR-125a-3p的靶基因。

IGF2位于11号染色体的短臂^[27]^，IGF2具有多种功能，参与细胞的增殖和分化过程。在*MyoD*基因诱导的心肌细胞分化早期IGF2表达水平上调^[29]^。当父系遗传导致IGF2缺失时，鼠胚胎继承的母系基因失活，而且会引起出生后侏儒^[29]^，提示IGF2在细胞分化、增殖以及胚胎发育中对母系基因激活具有重要作用。IGF2的表达上调还能够通过提高ERK1/2的磷酸化促进Wilms肿瘤的发生^[30]^。在血管生成方面，IGF2能够在肝细胞癌中上调VEGF的表达^[31]^，提示IGF2能够促进肿瘤发生以及肿瘤血管的形成。在胃癌的研究中发现，*IGF2*基因在胃癌的淋巴结转移中起了重要的作用^[32]^。在乳腺癌的研究中发现，IGF2表达能够增加乳腺癌恶变的可能性^[33]^。可见IGF2在多种肿瘤的进展中起着重要作用。

本研究应用miRNA芯片技术筛选了不同转移潜能人大细胞肺癌细胞系miRNAs的差异表达谱，发现22个差异表达的miRNAs。在高转移潜能人大细胞肺癌细胞系中，有13个上调和9个下调的miRNAs，其中miR-125a-3p的表达下调。靶点预测显示*IGF2*可能是miR-125a-3p的靶基因。在本研究的基础上，我们将对miRNA在肺癌转移中的作用和分子机制进行深入研究，为揭示miRNA调控肺癌转移的机制奠定基础，并为肺癌早期诊断和治疗提供新的靶点。
